# Identification of BDNF Sensitive Electrophysiological Markers of Synaptic Activity and Their Structural Correlates in Healthy Subjects Using a Genetic Approach Utilizing the Functional BDNF Val66Met Polymorphism

**DOI:** 10.1371/journal.pone.0095558

**Published:** 2014-04-23

**Authors:** Fruzsina Soltész, John Suckling, Phil Lawrence, Roger Tait, Cinly Ooi, Graham Bentley, Chris M. Dodds, Sam R. Miller, David R. Wille, Misha Byrne, Simon M. McHugh, Mark A. Bellgrove, Rodney J. Croft, Bai Lu, Edward T. Bullmore, Pradeep J. Nathan

**Affiliations:** 1 Clinical Unit Cambridge, GlaxoSmithKline, Cambridge, United Kingdom; 2 Brain Mapping Unit, Department of Psychiatry, University of Cambridge, United Kingdom; 3 Department of Psychology, University of Exeter, Exeter, United Kingdom; 4 Queensland Brain Institute, University of Queensland, Queensland, Australia; 5 School of Psychology and Psychiatry, Monash University, Melbourne, Australia; 6 Tsinghua University Medical School, Beijing, China; 7 New Medicines, UCB Pharma, Brussels, Belgium; Virginia Tech Carilion Research Institute, United States of America

## Abstract

Increasing evidence suggests that synaptic dysfunction is a core pathophysiological hallmark of neurodegenerative disorders. Brain-derived neurotropic factor (BDNF) is key synaptogenic molecule and targeting synaptic repair through modulation of BDNF signalling has been suggested as a potential drug discovery strategy. The development of such “synaptogenic” therapies depend on the availability of BDNF sensitive markers of synaptic function that could be utilized as biomarkers for examining target engagement or drug efficacy in humans. Here we have utilized the *BDNF* Val66Met genetic polymorphism to examine the effect of the polymorphism and genetic load (i.e. Met allele load) on electrophysiological (EEG) markers of synaptic activity and their structural (MRI) correlates. Sixty healthy adults were prospectively recruited into the three genetic groups (Val/Val, Val/Met, Met/Met). Subjects also underwent fMRI, tDCS/TMS, and cognitive assessments as part of a larger study. Overall, some of the EEG markers of synaptic activity and brain structure measured with MRI were the most sensitive markers of the polymorphism. Met carriers showed decreased oscillatory activity and synchrony in the neural network subserving error-processing, as measured during a flanker task (ERN); and showed increased slow-wave activity during resting. There was no evidence for a Met load effect on the EEG measures and the polymorphism had no effects on MMN and P300. Met carriers also showed reduced grey matter volume in the anterior cingulate and in the (left) prefrontal cortex. Furthermore, anterior cingulate grey matter volume, and oscillatory EEG power during the flanker task predicted subsequent behavioural adaptation, indicating a BDNF dependent link between brain structure, function and behaviour associated with error processing and monitoring. These findings suggest that EEG markers such as ERN and resting EEG could be used as BDNF sensitive functional markers in early clinical development to examine target engagement or drug related efficacy of synaptic repair therapies in humans.

## Introduction

The brain-derived neurotrophic factor (BDNF) and its receptor, tropomyosin-related kinase receptor type B (TrkB) are widely distributed in the human brain and play a significant role in supporting neuronal structure and function. In vitro experiments have shown that BDNF enhances synaptic transmission via multiple mechanisms. BDNF modulates long term potentiation (LTP) [1], [2], [3], and also promotes synaptic growth (i.e. synaptogenesis) and synaptic functioning by increasing spine density, axonal growth and branching, and facilitating the expression of the synaptic proteins synaptophysin, synaptobrevin and synaptotagmin [4], [5], [6]. BDNF enhances spatial learning and memory in rats [7], meanwhile pharmacologic and genetic deprivation of BDNF yields impairments in learning and memory performance in these animals [8]. Although BDNF is widely distributed in the human brain, its expression is reduced in neurodegenerative disorders including Alzheimer's disease, Huntington's disease and Parkinson disease [9], [10], [11], [12], [13], [14]. The possible role of BDNF in mood and psychiatric disorders, such as bipolar disorder and clinical depression, has also been indicated by several studies (for reviews see [15], [16], [17]). Therefore, therapeutic strategies aimed at synaptic repair and regeneration may be a viable strategy as disease-modifying treatment of neurodegenerative diseases (see review by [18]; [14], [16], [19]).

Synaptic degeneration is a core pathophysiological hallmark of neurodegenerative disorders. In Alzheimer's disease (AD), there is progressive synapse loss in the cortex and hippocampus [20], [21] and synapse loss has been shown to correlate with disease progression [22] and with episodic memory impairments [20]. These findings are supported by pre-clinical studies where age-dependent deficits in hippocampal long-term potentiation (LTP) and hippocampus-dependent memory have been reported in AD mouse models [23]. Beta-amyloid accumulation has been identified as a significant part of the pathogenesis of AD, leading to synaptic loss and memory impairments [24]. BDNF has been shown to exert neuro-protective effects against β-amyloid induced neurotoxicity both in vitro and in vivo in rats [25], and to ameliorate cognitive deficits caused by synaptic lesion in an animal model of dementia [26]. Further, Nagahara et al. [19] have demonstrated in different animal models including transgenic mice, aged rats and aged primates, that BDNF administration ameliorates behavioural and cognitive deficits by preventing cell death and neuronal atrophy in neuronal circuits involved in AD.

The development of “synaptogenic” therapies depend on the availability of BDNF sensitive markers of synaptic structure and function enabling synaptic dysfunction and repair/regeneration to be measured in clinical trials. A genetic variation in the human BDNF gene, a single nucleotide polymorphism (SNP) at nucleotide (G196A, rs6265) has been identified with in vitro experiments demonstrating that the G196A transition mutation in the coding region of BDNF results in a non-conservative amino acid substitution (valine [Val] to methionine [Met]) at codon 66 in the pro-domain of precursor BDNF protein. The Met variant is associated with impaired dendritic trafficking of BDNF, segregation into regulated secretory vesicles and synaptic localization, and decreased activity-dependent secretion of BDNF (18–30% decrease) [27], [28]. The Val66Met polymorphism has enabled investigation on potential markers of synaptic structure and function associated with changes in BDNF in humans. Although attempts to establish a direct link between the polymorphism and neurodegenerative diseases have been inconclusive [18], cross-sectional and longitudinal studies have demonstrated structural and functional differences between the phenotypes. Structural magnetic resonance imaging (MRI) have shown some evidence for reduction in grey matter volume in the hippocampus, amygdala and cortex in *BDNF* Met carriers [29], [30], [31], [32]. These findings are supported by a longitudinal study in healthy subjects showing greater age-related reductions in hippocampal volume in the *BDNF* Met allele carriers [33], and by a longitudinal study reporting greater cognitive decline over 36 months in subjects with high beta amyloid load carrying the Met allele [34]. This latter interaction between the Met allele and high beta amyloid load (a risk factor of Alzheimer's disease) suggests that the polymorphism, hence BDNF, might have an effect on disease progression. Functional magnetic resonance imaging (fMRI) studies have however yielded inconsistent findings. Some studies suggest that *BDNF* Met carriers exhibit reduced hippocampal activation during episodic memory encoding or retrieval compared with *BDNF* Val/Val subjects [27], [35], even when performance levels were matched [35], but when effects on successful memory related activation were examined, *BDNF* Met carriers showed a greater engagement of the hippocampus and additional areas in medial temporal lobe during encoding and retrieval, potentially suggesting neural inefficiency in memory-specific networks [36]. Similarly, studies examining synaptic activity using brain stimulation – methods such as transcranial magnetic stimulation (TMS), transcranial direct current stimulation (tDCS) or paired associative stimulation (PAS) – revealed inconsistent findings and sometimes paradoxical results regarding impairments in cortical excitability or plasticity in *BDNF* Met carriers [37], [38], [39], [40], [41], [42].

One potential explanation for the inconsistent findings across studies might be that in most of the cases, Met carriers including both Met/Met homozygote subjects and Val/Met heterozygotes are compared to Val/Val homozygotes [43]. The Met/Met genotype is relatively rare (<5%; [44]) and its occurrence might vary across studies. If the number of met alleles, i.e. the ‘met load’ exerts stepwise effects on neuronal functioning, then balancing the number of Met/Met subjects and Val/Met subjects is important. In fact, evidence from a recent pharmacogenetics study in mice suggests that the met/met homozygote variant yield significant disadvantages in synaptogenesis in the prefrontal cortex induced by the NMDA receptor antagonist ketamine, when compared to mice that carry at least one of the val allele [45]. Furthermore, stepwise decrease in performance on cognitive tasks tapping into intelligence, processing speed and memory recall has been found in elderly human subjects [46]. Therefore, in this present study, we investigate whether ‘met load’ yields any systematic differences in human neuronal activity underlying cognitive performance.

Electrophysiological methods measure the neuroelectric activity in the brain and hence are likely to be the most sensitive in vivo indicators of synaptic activity, neural network synchronization and function. Compared to studies utilizing structural and functional magnetic resonance imaging, relatively few studies have examined the effect of the Val66Met polymorphism on electrophysiological markers of neural activity and synaptic functioning. Resting electroencephalography (EEG) experiments revealed a general increase in slow-wave activity (theta and delta power) but a decrease in fast wave activity (alpha power) in *BDNF* Met carriers, suggesting an increase in inhibitory and/or a decrease in excitatory synaptic activity in the cortex [47], in accordance with earlier findings from in vitro and in vivo animal studies showing that BDNF regulates both excitatory and inhibitory synaptic functioning [5], [48]. Similarly, studies using event related potentials have also shown that *BDNF* Met carriers exhibit impairments in various cognitive paradigms probing error processing (i.e. decreased δ frequency band total power and synchronization during an error related negativity (ERN) task) [49] and attention (i.e. P300 latency increase and amplitude reduction) [29], [50].

As part of a larger investigation into the role of BDNF on synaptic and neural network activity in humans, we focused our investigation on electrophysiological markers including ERN, Mismatch Negativity (MMN), resting EEG activity/synchronization and P300. These EEG/ERP markers were selected because they have been shown to be sensitive to the *BDNF* Val66Met polymorphism [29], [49], [50], and/or impaired in neurodegenerative disorders including AD (e.g. [51], [52]). The ERN is an event related potential (ERN) produced in response to processing errors (i.e. when an event is worse than expected) [53], [54]. It is argued that the occurrence of a negative event, an error, elicits a dopaminergic error-signal from basal ganglia to the anterior cingulate cortex (ACC). The ACC then elicits the electrophysiological marker of error processing, the ERN [55], [56], [57], [58]. The ERN reflects increased neural synchronization in the error-processing network, which leads to the intensification of performance monitoring and behavioural adaptation [49], [59], [60]. MMN is an ERP that provides a neurophysiological representation of the pre-attentive acoustic change detection system (i.e. when the brain detects that an established pattern in sensory input has been violated) [61], [62]. It signals a change (i.e. prediction error signal) from what was expected (i.e. predicted) on the basis of the preceding auditory environment based on a memory representation [63], [64]. P300 is an ERP elicited in response to target (i.e. P3b) or novel (i.e. P3a) stimuli presented amongst standard stimuli in an oddball paradigm [65]. The P3a is thought to reflect an alerting process (i.e. focal attention) while, the P3b is thought to reflect context updating and working memory processes (for reviews see, [65], [66]). Resting EEG oscillations signify the intensity and synchrony of neural activity (i.e. EEG power) across various frequency bands. Fast wave oscillations (alpha frequency, ∼8–12 Hz) are associated with cortical excitatory mechanisms while slow-wave oscillations (delta, ∼0.5–3.5 Hz and theta frequency, ∼4–7 Hz) reflect cortical inhibition mechanisms; these mechanisms are maintained and balanced via homeostatic regulations within cortico-thalamical networks in the idling brain [67], [68].

Hence, the aim of the study was to examine the effect of the *BDNF* Val66Met polymorphism and the effect of *Met allele load* (i.e. the number of Met alleles) on changes in synaptic activity as measured by ERN, resting EEG oscillations, mismatch negativity (MMN), and P300. As the electrophysiological investigation was part of a larger study examining the effect of the polymorphism on other markers (i.e. hippocampal activity during declarative memory (fMRI), brain structure (MRI) and cortical excitability (Transcranial Direct Current Stimulation (tDCS)), we also briefly report these effects for comparison of effect sizes. The detailed findings on these markers will be reported and published elsewhere.

## Methods

### Ethics statement

The study was approved by Cambridge South National Research Ethics Committee (REC reference 11/EE/0360). Participants provided written consent to participate in the study. The consent procedure was approved by the Cambridge South National Research Ethics Committee.

### Subjects

Sixty healthy, right-handed volunteers participated in the study (39 males; mean age: 40.5 yrs, rage: 19–55 yrs). Twenty participants were homozygous for the met allele (Met/Met), 20 were heterozygous (Val/Met), and 20 were homozygous for the val allele (Val/Val). Subjects for the study were recruited from a database of approximately 10,000 subjects with information on the *BDNF* gene polymorphism, held at the Phase I GSK Clinical Unit and the Cambridge BioResource, Cambridge Biomedical Research Centre (CBRC). Level of education across the three groups is reported in Table S1 (File S1). Level of education was comparable across the three groups. Participants went through a thorough screening procedure before entering the study, whereby subjects with a history of Axis I psychiatric disorders, neurological disorders, any medical condition or illness affecting their participation, alcohol or substance abuse, pregnancy, were excluded from study participation (for more details on recruitment, exclusion criteria, and procedure see [69]). Participants were also non-smokers and free of any medication.

### Genotyping

DNA was extracted from blood samples via standard methods and genotyped for the *BDNF* Val66Met SNP via TaqMan 50exonuclease assay (Applied Biosystems, Foster City, CA, USA) (described in more detail in Teo et al., [86]). Subjects were all Caucasian except one who was of mixed ethnicity. The genotypes in this sample are not in Hardy-Weinberg Equilibrium. This was intentional – an equal number of subjects was recruited from each of the genotype groups in order to look at gene-dose effects in a balanced design.

### Procedure

After a through screening session selecting volunteers eligible for the study, volunteers attended two separate testing sessions on two separate days. EEG and MRI measurements were performed as part of a wider study examining a variety of neurophysiological and behavioural endpoints, but only the EEG and the structural MRI data are reported here in detail. Subjects arrived at the GSK Clinical Unit in the morning and a urine drug screen and alcohol breath test was performed followed by EEG assessment. MRI scanning was performed between 12.30 and 14.30 hours. The study was double-blind.

### Experimental paradigms

#### Error-related negativity (ERN)

The flanker task, an established paradigm for exploring the behavioural and neural correlates of error processing [70], [71], was used to generate the ERN.

**Figure S1** illustrates the task procedure and stimuli. Participants were asked to indicate the direction of the target arrow (middle) as soon as possible after it has appeared between the two flanker arrows. Flanker and target can be congruent (i.e. flankers pointing into the target's direction), incongruent (flankers point to the opposite direction), and neutral. Subjects tend to commit more errors on incongruent trials and also tend to slow down after an incorrect response (‘post-error slowing’; [72]). Recent accounts of post-error slowing hold that it reflects attentional orientation associated with violation of expectations [72], and with behavioural adaptation processes [49], [73]. The magnitude of post-error slowing is thought to index the strength of the error monitoring response [74]. The electrophysiological marker of error processing, the error-related negativity (ERN; [54]) is observed when response-locked ERPs for error trials are compared to correct trials.

Stimuli were presented using Neuroscan Stim^2^ system with visual stimuli delivered to a CRT monitoring a darkened room. Participants seated comfortably in front of a 75 Hz, 17" CRT monitor positioned approximatelly 95 cm away from their eyes. Participants were asked to indicate the direction of the target (middle) arrow as fast as possible with a button press using their left or right thumb. Reaction times (RT) and error rates were recorded.

Error rate (%) was calculated as the ratio of erroneous responses. Post-error slowing (PES), an index of error processing [49], was calculated as the difference between RTs following erroneous responses and RTs following correct responses. PES was also corrected for general speed (PES divided by mean reaction time); so that possible differences in general speed between groups would not yield group differences in PES.

#### Resting state EEG

Approximately 7.5 minutes of EEG data were recorded during resting, with eyes closed. Participants were asked to close their eyes and to relax but to not move or fall asleep.

#### Target P3 (P3b) and Novelty P3 (P3a)

An “oddball” paradigm [75] with three different tones was used to elicit the P300 and P3a ERP components. The ‘standard’ stimuli were 1000 Hz tones occurring with 85% probability. The ‘target’ stimuli were 2000 Hz tones occurring with 7.5% probability. The ‘novel’ stimuli were white noise, occurring with 7.5% probability. The ISI ranged between 1000 and 2200 ms, randomised in 100 ms steps. Subjects were asked to listen to the tones and indicate with a button press when they heard the ‘target’ (but ignore the ‘novel’) sound. Tones were played binaurally through ear inserts at 80 dB.

#### Mismatch negativity (MMN)

Subjects were seated comfortably in front of a CRT monitor and asked to watch scenes from a wildlife documentary without sound. Tones were played binaurally through ear inserts at 80 dB. Duration deviants with roving frequency design were used to generate MMN. Standard tones were 50 ms long and deviant tones were 100 ms long, with the latter presented at the end of a train of standard tones of 12–15 in length. Tones were separated by an ISI of 350–450 ms, randomised in 10 ms steps. Between each train of standard/deviant stimuli, one to two new mask tones were presented. Each train was presented at a different frequency, separated from the previous train by at least 500 Hz (tone range 500–5000 Hz).

### EEG recording and analysis

EEG was recorded using a Neuroscan Synamps^2^ EEG amplifier using a 64 channel QuickCap with integrated sintered Ag/AgCl electrodes, reference electrode located between Cz and CPz. Additional individual electrode wires were placed bilaterally on the outer canthi and above and below the left eye to record the EOG. An individual electrode wire was placed on the nose to use as a re-reference electrode for the MMN paradigm. Electrode impedances were below 10 kOhms at the start of the recording. Bioelectric signals were amplified in AC mode with a 24 bit ADC providing 3nV/bit resolution. EEG was recorded with 0.3 Hz high pass and 200 Hz anti-aliasing filters and sampled at 1000 s/s. Responses were captured through a 4 button response pad (part of the STIM2 system) with a 1 ms response accuracy.

Data pre-processing and artefact rejection were performed offline using Neuroscan Curry7 Neuroimaging Suite. Eye movement correction was performed by PCA. Epochs containing activity ±100 µV were excluded from further analyses. Six subjects' data were discarded from the ERN analysis (two from each genetic group) because after artefact rejection there were only a low number of error trials (<5%) in their data. Further EEG analyses were carried out in MatLab (MathWorks).

#### Error-related negativity, time-domain analysis

Offline data were re-referenced to the average electrode and a second-order lowpass (80 Hz) Butterworth filter (corresponding to 12 dB/octave rolloff) was applied. For the time-domain ERP analysis the data was further smoothed with a 51 point long moving average filter in order to remove high-frequency noise from the ERP waves. This filtering did not affect the frequency spectrum for time-frequency analyses. Data were then epoched into 3000 ms long segments time-locked to responses (−1500 ms to +1500 ms around error or correct response markers at time point zero). Epochs were baseline corrected to the −800 to −600 ms interval [49]. Time-domain ERPs were then truncated to the −800 to 800 ms interval. Event-related potentials (ERPs) evoked by correct responses were averaged together within each subject to create the correct response negativity (cN). Similarly, ERPs evoked by error trials were averaged together to create the error-related negativity (ERN). Difference waves were created by subtracting the correct from the erroneous waveforms within each participant. Following the visual inspection of the mean ERPs, peak amplitudes and peak latencies of cN, ERN, and difference waves were automatically detected within the 0–200 ms post-stimulus window. Data from electrode FCz is presented and submitted to further analysis, since the effect was the strongest at this location (see the time-domain ERP results and also [49]).

#### Error-related negativity, time-frequency analysis

Following up the time-domain ERN analysis, time-frequency power and phase-locking (PL) analyses were performed on all trials (i.e. trial-by-trial analysis) using short-time Fourier transforms with Hanning window tapering, resulting in a time-frequency landscape with a resolution of ∼3 ms in time and 0.49 Hz (from 0.5 to 30 Hz) in frequency. For the time-frequency analysis scripts from the EEGLAB toolbox [76] were used. Three thousand millisecond-long (3000 datapoints) epochs were used for the decomposition. Based on the topographical location of the time-domain ERN, and following earlier findings in the literature [49], data from the FCz electrode were analysed and presented.

#### Resting state EEG

EEG was epoched into approximately 4 second segments (4096 samples).

Data were transformed using fast Fourier Transform. Average power in the defined frequency bands of 0.5–3.5 (delta), 4–7.5 (theta), 8–11.5 (alpha), 12–29.5 (beta), 30–80 Hz (gamma) and 0.5–80 Hz as total power across all bands were calculated.

Data from individual electrodes were grouped according to topographical regions based on their anatomical location and were averaged together and were analysed as whole regions. Regions were Frontal (Fp1, Fpz, Fp2, AF3, AF4, F7, F5, F3, F1, Fz, F2, F4, F6, F8), Central (FC3, FC1, FCz, FC4, C3, C1, Cz, C2, C4, CP3, CP1, CPz, CP2, CP4), Temporal (T7, TP7, P7, T8, TP8, P8) and Parieto-occipital leads (P3, P1, Pz, P2, P4, PO3, POz, PO4, O1, Oz, O2) were averaged together and were analysed as whole regions.

#### Target P3 (P3b) and Novelty P3 (P3a)

Offline data were re-referenced to the linked mastoids and a second-order lowpass (30 Hz) Butterworth filter (corresponding to 12 dB/octave rolloff) was applied. Data were then segmented into 900 ms long epochs, time-locked to the stimuli (100 ms before stimuli and 800 ms after stimuli). Epochs were baseline-corrected to the −100 to 0 ms interval. ERPs evoked by ‘target’ tones were averaged together to form the P3b ERP component and ERPs evoked by ‘novel’ stimuli were averaged together to form the P3a ERP component. Peak amplitude and latency of the P3b within the time window of 200–800 ms was measured at the CPz electrode. Peak amplitude and latency of the P3a within the time window of 200–500 ms was measured at the Cz electrode. Time windows and examined electrodes were selected based upon where these peaks are the most prominent [65].

#### Mismatch negativity (MMN)

Data was re-referenced to the nose electrode. Epochs of −100 ms to 500 ms time-locked to stimulus presentation were extracted. Epochs were baseline-corrected to the −100 to 0 ms interval. Responses to standard stimuli at all train positions (with the exception of position 1) were averaged together to create the standard average wave. Responses to deviant stimuli were averaged to create the average deviant wave. The standard was then subtracted from the deviant response to create the difference waveform. Data from the Fz electrode was analysed [77].

### Statistical analyses

Group differences, experimental condition effects (in the flanker task), and their potential interactions were tested with analysis of variance (ANOVA). Age and gender were entered as covariates to the analyses. Post-hoc pairwise comparisons, corrected for multiple comparisons (Tukey-Kramer correction) were performed when appropriate. For group comparisons, two different genetic models were considered: (1) the met-dominant model, where Met carriers are grouped together and compared against Val/Val homozygotes (this model is the most prominent in the literature); and the (2) additive model, where the three genotype groups were entered into ANOVA separately in order to test whether there were stepwise effects due to ‘met load’. Outliers, who fell outside three standard deviations from the mean, were excluded from further analyses. All the relevant variables were normally distributed (according to the Kolmogorov-Smirnov test, all p>0.2). Only behavioural error rates and the latency of the ERN peak following correct responses were not normally distributed according to the test. Fisher's z-transform was applied to the error rate data. Latencies after correct responses were not relevant for the analysis. Effect sizes (partial eta squared) for the comparability of different measures and platforms are also reported. We report partial eta because this measure is one of the most common measures of effect size used in factorial ANOVA designs and also recommended in neuroscience research [78].

Primarily, we report p-values for each measure uncorrected for multiplicity. In addition, for each platform where at least one p<0.05 was observed, we applied the false discovery rate (FDR) procedure as described by Benjamini and Yekutieli to allow for dependency between test statistics [79], to confirm the FDR within each platform was controlled at 5%.

### Structural MRI data acquisition and pre-processing

MR images were acquired on a 3T Siemens TimTrio at the Wolfson Brain Imaging Centre, University of Cambridge. Structural data were acquired with a sagittal MPRAGE T1-weighted, three-dimensional, inversion recovery gradient echo sequence with the following parameters: Inversion time  = 900 ms; echo time  = 2.98 ms; repetition time  = 2300 ms flip angle  = 9 degrees; voxel dimensions  = 1 mm×1 mm×1 mm. Acquisition time  = 9.14 mins.

Structural data was analysed with FSL-VBM [80], an optimised VBM protocol [81] carried out with FSL tools [82]. Firstly, structural images were reoriented to standard space, brain-extracted and grey matter-segmented before being aligned to the MNI 152 template [83]. MNI 152 alignment involved affine registration of grey matter images to GM ICBM-152 to create a first pass affine template; non-linear re-registration of native grey matter images to the affine template was then performed. Resulting warped images were averaged and flipped along the x-axis in order to create a left-right symmetric, study-specific grey matter template. To avoid bias in the form of favouring one group over another during registration, all subjects from contrasting groups were included in the template construction process. Secondly, all native grey matter images were non-linearly registered to the study-specific template and modulated to correct for local expansion and contraction due to the non-linear component of the spatial transformation. Modulation was achieved by multiplication of each voxel of each registered grey matter image by the Jacobian of the associated warp field. Finally, modulated grey matter images were smoothed with an isotropic Gaussian kernel with a sigma of 3 mm. To calculate ACC and PFC volumes, the AAL atlas was employed as a mask for summing then averaging underlying warped and modulated grey matter image intensities.

#### Statistical analysis of structural MRI data

Voxel-wise GLM statistical analysis was performed across the whole-brain using CamBA v2.3.0 (http://www-bmu.psychiatry.cam.ac.uk). The location of clusters of interest was established using CamBA derived spatial information. The independent variable was a linear contrast across met-loading or a met-dominant model as described above. Sex, age and total intracranial grey matter volume were added as regressors. Estimates of the linear effect size divided by its associated standard error were tested for significance using a permutation method at the cluster level, correcting for multiple comparisons across clusters. All results are reported at a significance level of <1 false positive cluster per image [84]. Using significant voxel clusters as masks, mean cluster intensities were extracted from modulated standard space grey matter images for all subjects then correlated with behavioural data using SPSS (v17).

## Results

### Demographics

There were no significant age or gender differences among the genetic groups (age: F(2,57) = 0.5, p>0.6, gender: H(2, N = 60) = 0.9, p>0.6. Mean age: 41.8, 40.5, and 38.5 years; males: 10, 15, and 14, for Met/Met, Val/Met, and Val/Val respectively. Group differences in age and gender remained non-significant after removing 6 subjects' data from the ERN analysis, both p>0.3).

### Behavioural results and error-related negativity in the flanker task

#### Behavioural data – Reaction times (RT)

Correct and error RTs were entered into a two-way mixed design ANOVA with Response Type (Correct vs. Error) as within-subject and Group as between-subject factor. Correct RTs were significantly slower than erroneous RTs (427.8 ms, SE: 3.9 and 355.4, SE: 3.9 for correct and error RTs, respectively. F(1,58) = 10.76, p = 0.001, η = 0.39). There was no main effect of group (both genetic models p>0.3) and no interaction of response type and group (all p>0.15).

#### Behavioural data – Error rates (%)

Error rates in congruent and incongruent conditions were entered into a two-way mixed design ANOVA with Condition as within-subject and Group as between-subject factor. The main effect of Condition was significant (0.8%, SE: 0.12 and 13.7%, SE: 1.5, for congruent and incongruent conditions, respectively. F(1,58) = 13.83, p<0.001, η = 0.44). Examination of the different genetic models did not yield significant differences in error rates (both p>0.26) or the modulation of the Condition effect by group (both p>0.18).

#### Behavioural data – Post-error slowing

**Figure 1:**
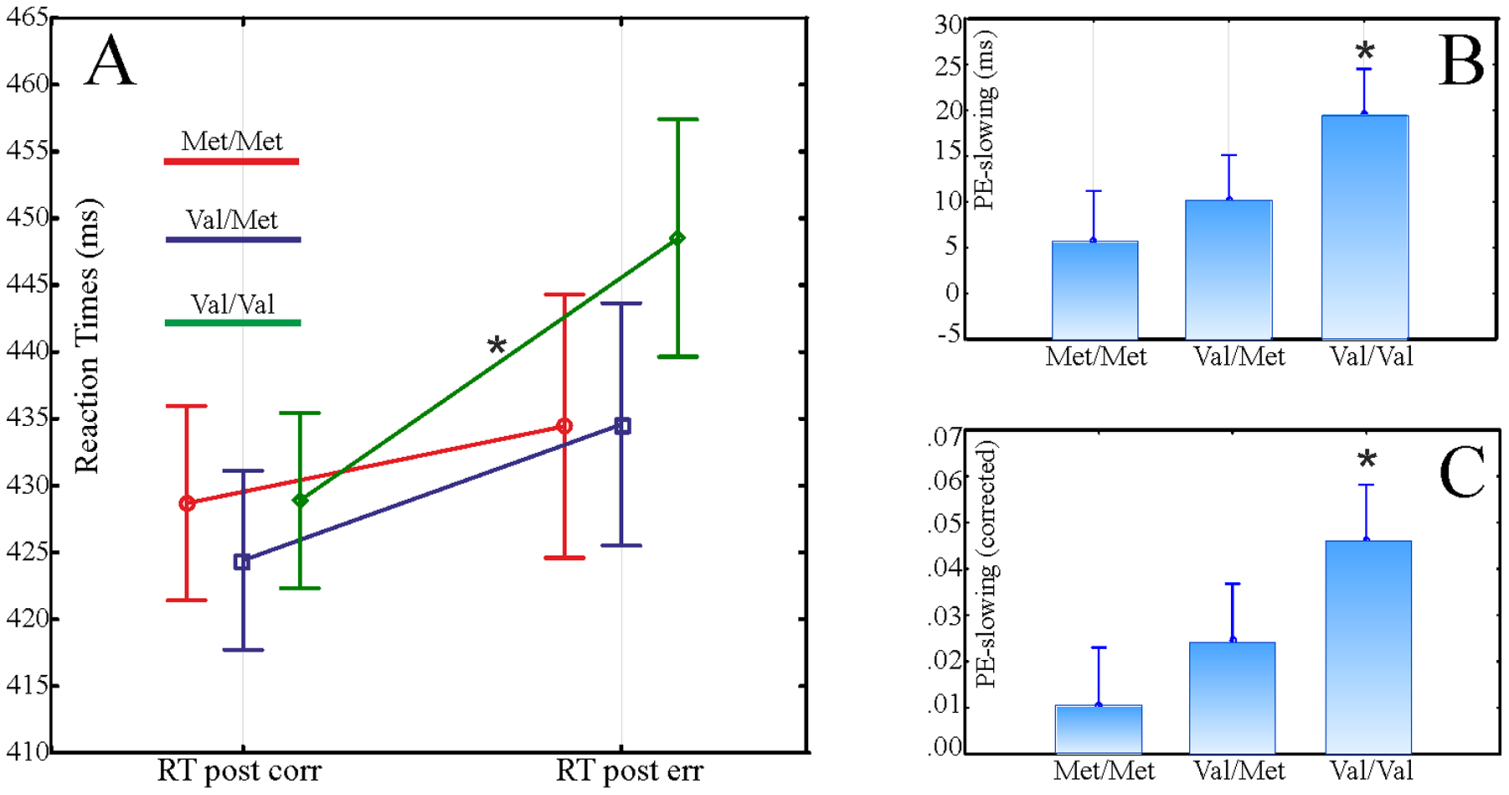
Behavioural results of the flanker task. Panel A: Raw RTs following correct and error responses for the three genetic groups. Panel B: Post-error slowing expressed in raw RTs (RT following error minus RT following correct responses). Panel C: Post-error slowing, corrected for general speed ([RT following error - RT following correct]/meanRT). *: p<0.05. Bars represent standard error.

Post-error slowing values were entered into a one-way ANOVA with Group as between-subject factor. Both the linear model (F(2,57) = 2.27, p = 0.038, η = 0.26) and the met-dominant model were significant (F(1,58) = 3.99, p = 0.05, η = 0.25). The post-error slowing is shown in Figure 1/A. The linear model best explained the data.

In order to investigate the source of the post-error slowing, post-hoc pairwise comparisons were performed on RTs following correct and erroneous responses within and across genetic groups. As **Figure 1** **/B** shows, the post-error slowing (i.e. the difference between post-correct and post-error RTs) was significant only in the Val/Val group (p = 0.004), while it was not significant in the Val/Met (p = 0.39) and in the Met/Met (p = 0.71) groups.

#### Time-domain ERP data

The amplitude and latency of the error-effect, the error-related negativity (ERN) expressed as the difference between error and correct responses, were subjected to a one-way ANOVA with Group as between-subject factor. Significant group differences were further explored using post-hoc comparisons among correct and error responses within and between genetic groups (corrected for multiple testing) are performed to investigate the source of the ERN.

**Figure 2:**
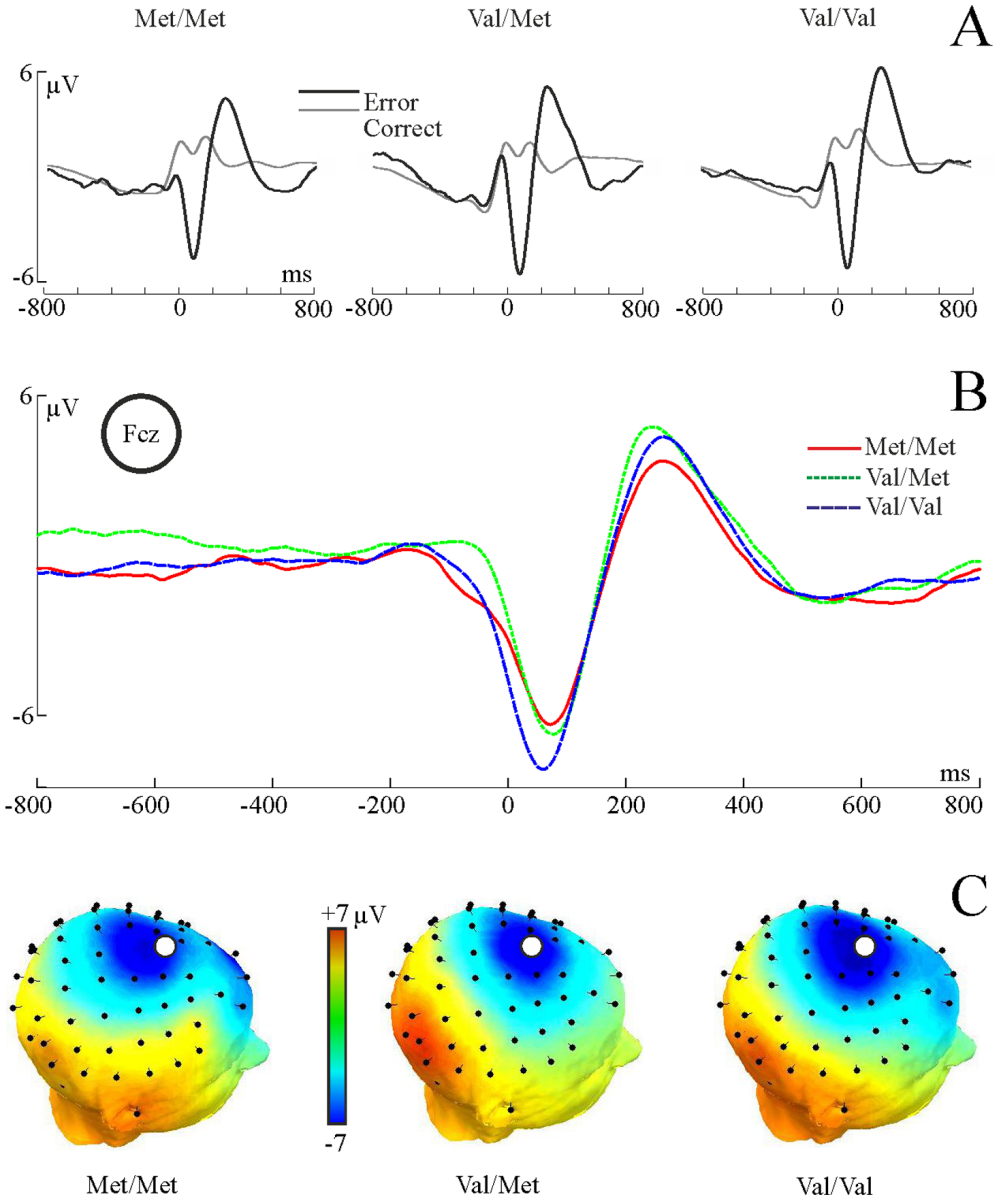
Time-domain ERN results. Panel A: Waveforms for correct (grey) and error (black) responses (FCz), for each group. Panel B: ERN wave for the three genetic groups. Panel C: Topographic plots of the ERN peak (40–100 ms). Electrode FCz is marked with disk.

Neither the ERN peak amplitude nor the ERN peak latency was significantly different among the genetic groups; neither of the genetic models were significant (both p>0.27). In order to make sure that the differences in referencing would cause different results, laplacian transformation (following Beste et al. 2010 [49]) has also been performed on the data. Neither of the genetic models were significant on the transformed data (both models' p>0.8; mean and SE: −50.62 (10.3) µV/m^2^, −58.7 (9.98) µV/m^2^, and −59.3 (30.86) µV/m^2^ for Met/Met, Val/Met and Val/Val groups respectively). Waveforms for error and correct responses and their difference wave (ERN) per groups are illustrated in **Figure 2**.

#### Time-frequency domain data

Based on the localization of the ERN component (see Figure 2) and on previous literature [49], time-frequency data from the FCz electrode were analysed. Mean ERSP peak values and PLF values of the difference spectrum in the delta (0.5–4 Hz) frequency band within the post-response time interval (0–250 ms) were subjected to a one-way ANOVA with Group as between-subject factor. The time-frequency ‘region of interest’ was a priori defined based on visual inspection of the data; which range approximately corresponds to earlier findings [49]. Significant group effects were further explored using pairwise comparisons (correcting for multiple testing) between correct and error responses within and across genetic groups.

#### Event-related spectral perturbation (ERSP) (i.e. Power)

Examination of the genetic models yielded a significant met-dominant model (F(1,52) = 4.5, p = 0.038, η = 0.29) (the linear model did not reach significance: p = 0.1).

**Figure 3:**
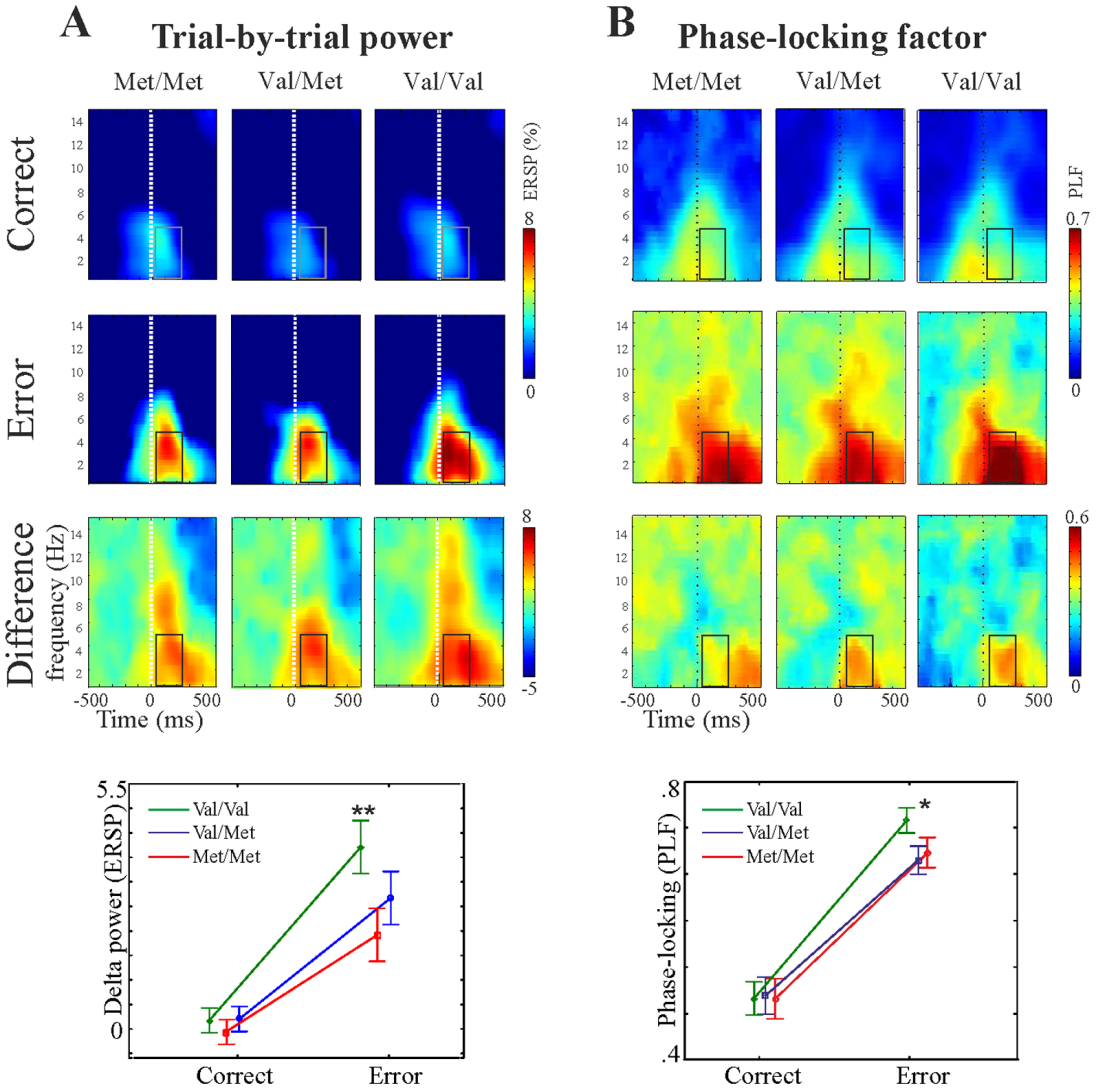
Behavioural results of the flanker task. Panel A: Event-related spectral perturbation (ERSP) during correct and error responses, and their difference (error-correct) for the three genetic groups. Boxes in the time-frequency plots indicate the time-frequency interval from which the values have been submitted to statistical analyses. Statistics are illustrated in the lower graph, bars represent standard error, **:p<0.005. Panel B: Phase-locking factor (PLF) values, as above. *:p<0.05.

Pairwise comparisons (corrected for multiple testing) of response type (correct and error) within and between genotype groups indicated that the above effect was due to significantly different error processing between Met carriers and Val/Val group. As can be seen in **Figure 3** **/A**, the effect of response type (i.e. correct versus error ERSP) was significant in both the Met carriers and in the Val/Val groups (p<0.001 for both). However, ERSP following error responses in the Val/Val group was significantly larger than ERSP following error responses in the Met carriers (p<0.001; Mean and standard error: 4.2 (0.54), 2.8 (0.4) for Val/Val and Met carriers, respectively). There were no difference in correct ERSP between the two groups (p = 0.99), indicating that the group differences were due to lower ERSP specifically in the error condition in met carriers.

#### Phase-locking factor (PLF)

Similar to the ERSP data, examination of the different genetic models yielded a marginally significant met-dominant model in the difference PLF (error-correct) (F(1,52) = 3.72, p = 0.059, η = 0.26; the linear model was not significant: p>0.15), indicating that the error-effect is modulated by the polymorphism (see Figure 3/B). Pairwise comparisons (corrected for multiple testing) of PLF following correct and PLF following error responses between and within Met carriers and Val/Val subjects revealed a significant effect of response type in both groups (correct versus error: both p<0.0002). Similar to the ERSP results, the group difference in the error-related PLF was due to significant differences specifically in the error condition. Val/Val participants showed significantly larger phase-locking (mean and standard error: 0.78 (0.03) following error responses compared to Met carriers (0.65 (0.026)) (p<0.005), while there was no group difference between groups in PLF following correct responses (p = 0.98).

### Grey matter volume

#### Anterior Cingulate and prefrontal cortex

**Figure 4:**
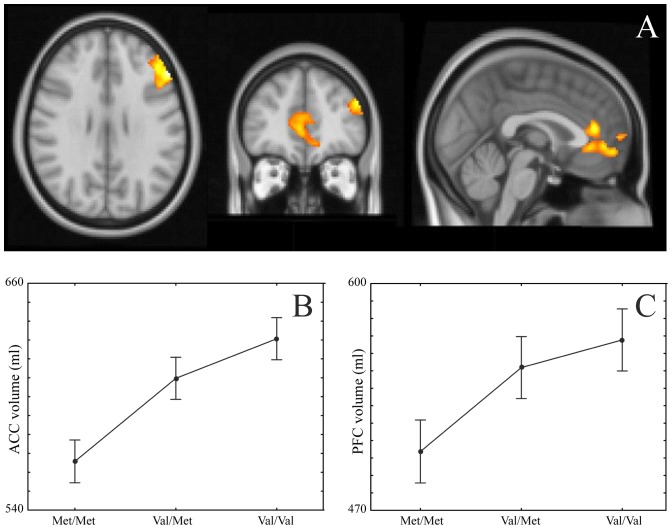
Anterior cingulate and prefrontal cortex grey matter volumes. Panel A: Met/Met vs Val/Val contrast. Significant cluster are rendered onto an average brain in MNI standard space. Panel B: Extracted anterior cingulate grey matter volume for each group. Panel C: Extracted prefrontal cortex (left) grey matter volume for each group. Bars represent standard error.

Examination of the different genetic models revealed significant whole-brain tested differences in grey matter volume located around the ACC, with the linear model explaining the data slightly better (linear: F(2,55) = 8.43, p<0.001, η = 0.48; met-dominant: F(1,56) = 8.4, p<0.005, η = 0.31 from data extracted from significant clusters). Further, a significant PFC GM volume loss has also been found in Met carriers (linear: F(2,55) = 3.34, p = 0.04, η = 0.1; met-dominant: F(1,56) = 3.02, p = 0.087, η = 0.22 again from data extracted from significant clusters). Figure 4 illustrates the group differences in grey matter volume.

### Correlations among error-related processes and Anterior cingulate grey matter volume

In order to reveal structural-functional relationships among behavioural and electrophysiological measures of error-processing and brain structure, endpoints showing significant genotype effects were subjected to correlational analyses. We were interested in the relationships among brain structure (grey matter volume), brain function and neuronal activity measured by ERSP and PLF, and behavioural function measured by post-error slowing. The results are summarized in **Table 1**. First, correlations between post-error slowing and 1) anterior cingulate grey matter volume, 2) prefrontal cortex grey matter volume, 3) ERSP following error responses, and 4) PLF after error responses were investigated. Second, correlations between anterior cingulate grey matter volume and 1) ERSP following error, and 2) PLF following error were investigated. Third, correlations between prefrontal cortex grey matter volume and 1) ERSP following error, and 2) PLF following error were tested.

**Table 1:** Correlations.

Var 1	Var 2	Met/Met	Val/Met	Met-carriers	Val/Val
**PE slowing**	**ACC GM**	***r = 0.67***	r = 0.16	***r = 0.36***	r = −0.36
		***p = 0.003*** **	p = 0.51	***p = 0.03*** *	p = 0.15
	**PFC GM**	r = 0.14	r = 0.03	r = 0.07	r = −0.26
		P = 0.59	p = 0.91	p = 0.68	p = 0.29
	**ERSP error**	r = 0.22	***r = 0.49***	***r = 0.38***	r = 0.29
		p = 0.4	***p = 0.054°***	***p = 0.027*** *	p = 0.28
	**PLF error**	r = −0.06	r = 0.06	r = 0.097	r = 0.25
		p = 0.83	p = 0.8	p = 0.69	p = 0.34
**ACC GM**	**ERSP error**	r = 0.34	r = 0.25	r = 0.11	r = −0.1
		p = 0.19	p = 0.35	p = 0.54	p = 0.7
	**PLF error**	r = −0.18	r = 0.08	r = −0.08	r = −0.15
		p = 0.53	p = 0.77	p = 0.72	p = 0.56

Columns Var1 and Var2 contain the two variables correlated with each other in the given line. First section: Var1 is Post-error slowing. Second section: Var1 is anterior cingulate grey matter volume. R-values and corresponding p-values are reported for the three genetic groups and for the met-carriers combined. Significant correlations are marked with bold italic typesetting.

°:p<0.06,

*:p<0.05;

**p<0.005.

**Figure 5:**
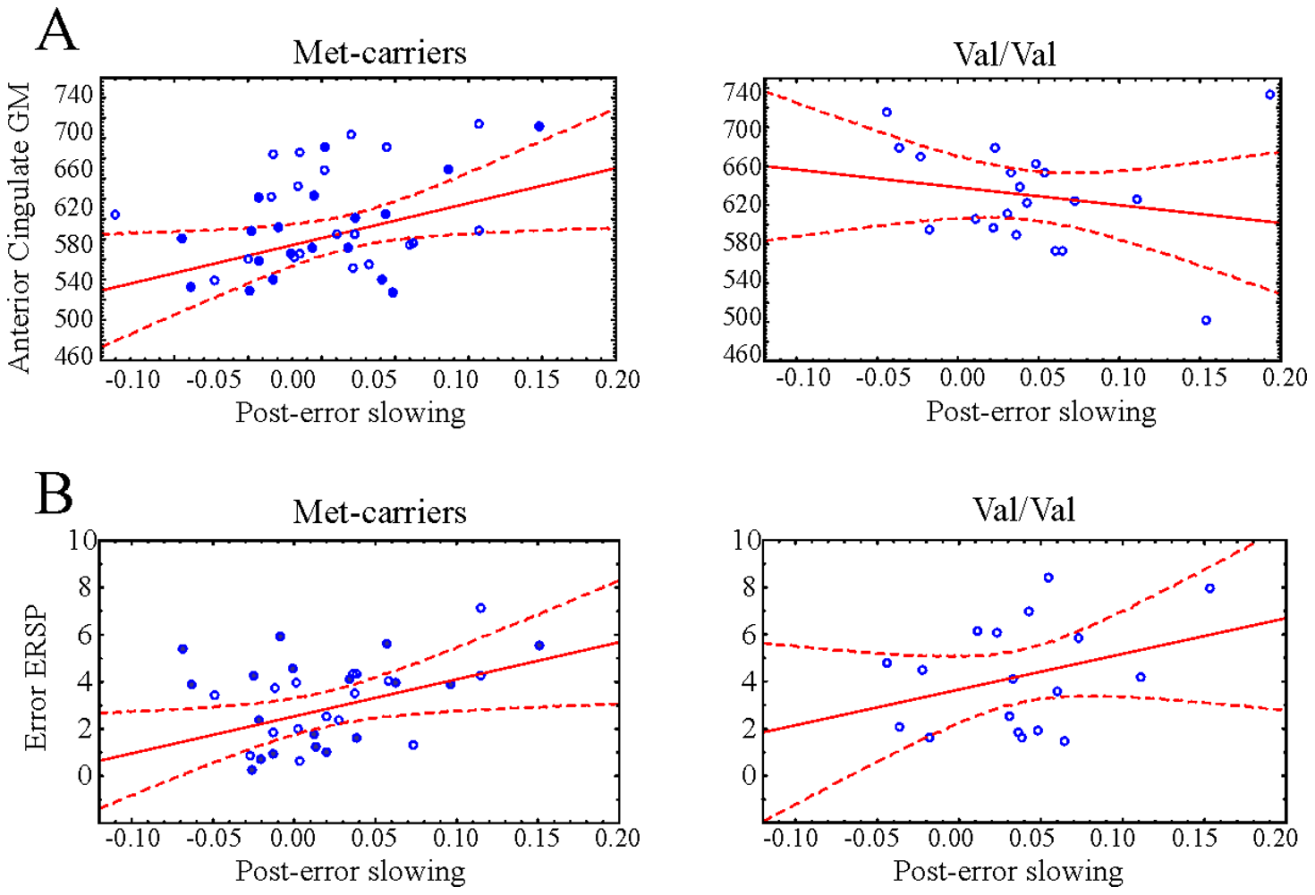
Correlations between grey matter and post-error slowing and between grey matter and ERSP. Panel A: Correlation between anterior cingulate grey matter volume and post-error slowing for met-carriers and Val/Val group. Met/Met subjects are filled dots, Val/Met subjects are empty dots in the Met-carriers plot. Dashed line indicates 95% confidence interval of the linear regression. Panel B: Correlation between error ERSP and post-error slowing, as above.

Post-error slowing showed significant correlations with anterior cingulate grey matter volume in Met/Met subjects but not in the other two groups (Val/Met and Val/Val); the larger the ACC volume, the larger the post-error slowing in Met/Met subjects. Furthermore, ERSP following error responses also significantly predicted post-error slowing in the Met carriers but not in Val/Val subjects. The significant correlations are shown in **Figure 5**.

Correlation coefficients were compared in order to see whether beyond the significance of the relationships, the strengths of the above indicated relationships are statistically different between the groups [85], [86]. The correlation between ACC grey matter volume and post-error slowing was indeed significantly stronger in Met carriers than in Val/Val participants (z-score = 2.097, p<0.036). For the correlation between ERSP and post-error slowing, although the ERSP was significantly predictive of post-error slowing in Met carriers but not in Val/Val subjects, the difference of the correlation values were not significant (z-score = 0.28, p>0.7). It indicates that the nature of the relationship between delta power and post-error slowing are not different, but somewhat stronger in Met carriers.

### Resting state EEG

#### Absolute power

**Figure 6:**
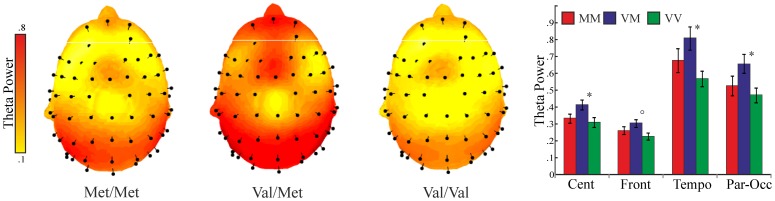
Topographic plots of power in the theta frequency band. Graph represents averaged values for central, frontal, temporal and parieto-occipital regions, per each genetic group. Bars represent standard error. * p<0.05;° p<0.06 according to post-hoc Tukey-Kramer comparisons (Val/Met vs Val/Val).

Examination of the different genetic models revealed significant group differences in the theta (4–7 Hz) frequency band during resting state. The met-dominant model explained the data significantly over frontal, central, temporal and parieto-occipital regions (all Fs(1,55)>4.9, p<0.03, η<0.28). Met carriers, especially the Val/Met group, showed increased activity in the theta band, compared to Val/Val subjects. The group differences are illustrated in Figure 6.

No significant group effects emerged in the other (delta, alpha, beta, gamma) frequency bands (all p>0.07). Results for all bands and regions are summarized in **Table S2** (File S1).

#### Relative power

The analysis of relative power, correcting for the possible general, total power differences among groups, confirmed the significant met-dominant model in the theta power (parieto-occipital: F(1,55) = 6.1, p<0.02, η = 0.32; and strong statistical trend for frontal, central and parietal regions: all p<0.07, η>0.22). No other significant differences emerged in the other frequency bands (all p>0.07).

#### P300 and MMN

The analysis of P3a and P3b ERP components, and of the MMN, yielded non-significant results. The MMN, P300 and P3a peak amplitudes and latencies are presented in Table S3 (File S1).

**Figure 7:**
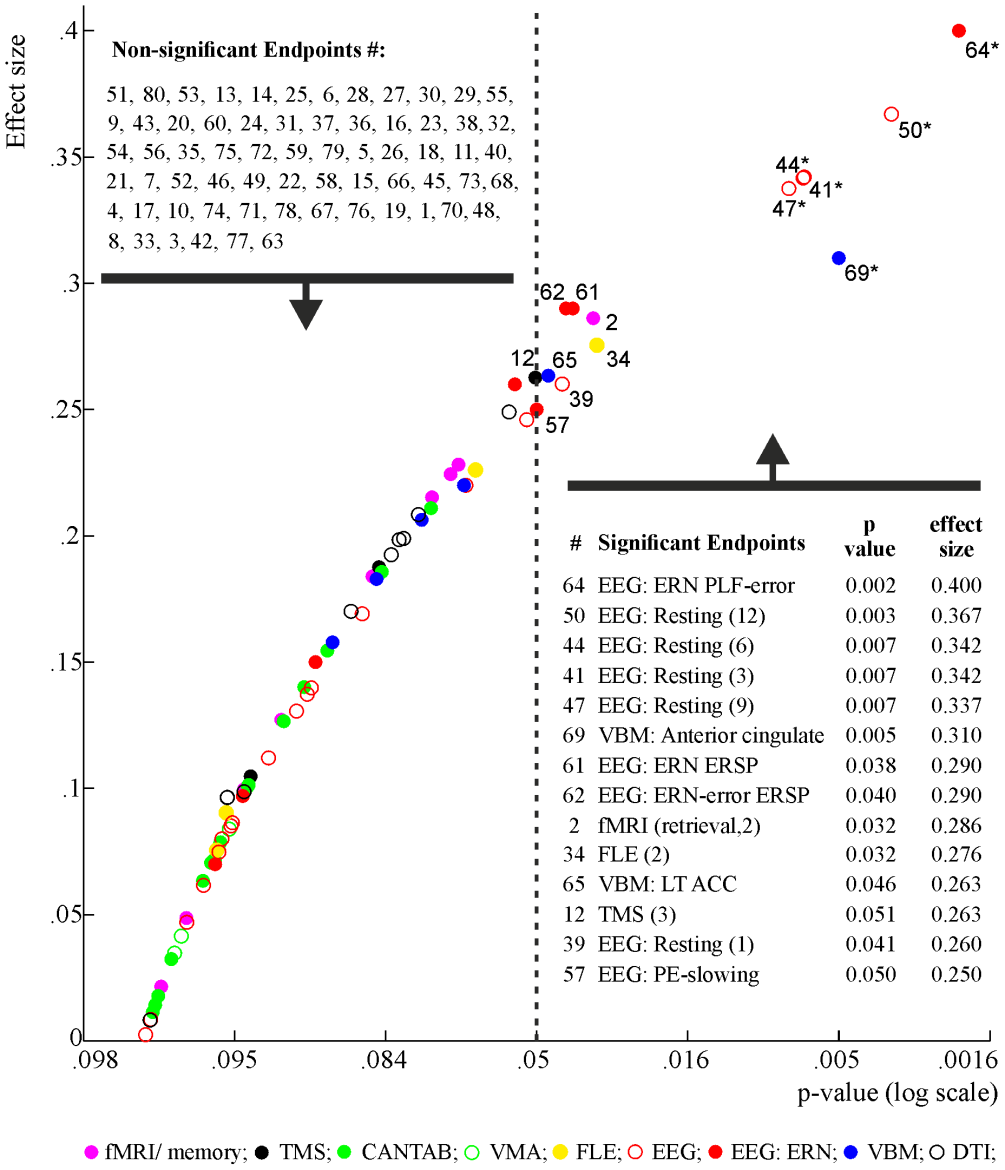
Effect sizes. P-values (log scale) (X-axis) and corresponding effect sizes (Y-axis) for the met-dominant model for every endpoint. Dotted vertical line marks the alpha level (0.05): endpoints falling on the right side are considered to be statistically significant (i.e. p<0.05). (*) indicates significant endpoints after FDR correction (all adjusted p<0.04). Different platforms are marked with different colours, for further details see Table 2. P-value transform: - log10(*p*).

In the study, endpoints from the other different platforms (fMRI, TMS, cognitive tests) have been analysed and reported in detail elsewhere [69], [87]. In order to illustrate the distribution of endpoints across all platforms, effect sizes and corresponding p-values for all the endpoints are shown in **Figure 7** (and **Table 2**). As can be seen in **Figure 7**, a few of the EEG endpoints and anterior cingulate grey matter form a cluster in the upper end of the distribution, indicating that these endpoints were sensitive to the polymorphism. In addition, for each platform where at least one p<0.05 was observed, we applied the false discovery rate (FDR) procedure [79], to confirm the FDR within each platform was controlled at 5%. Endpoints remaining significant after the FDR correction are indicated in **Figure 7**. Namely, endpoints with p<0.008 remain significant after the correction (phase-locking following errors in the theta band, EEG resting state power, and anterior cingulate grey matter volume).

**Table 2:** Effect sizes for the endpoints across all platforms.

Platform	#	Endpoint	Description	effect size
fMRI/memory	1	fMRI (retrieval,1)	Ret: Hits v Correct rejections (Left hippocampus)	0.215
	2	fMRI (retrieval,2)	Ret: Hits v Correct rejections (Right hippocampus)	0.286
	3	fMRI (retrieval,3)	Ret: Hits v Misses (L HC)	0.228
	4	fMRI (retrieval,4)	Ret: Hits v Misses (R HC)	0.184
	5	fMRI (encoding,1)	Enc: Hits v Misses (L HC)	0.100
	6	fMRI (encoding,2)	Enc: Hits v Misses (R HC)	0.022
	7	Memory (d-prime)	d-prime	0.127
	8	Memory (Hits)	Hits (%)	0.224
	9	Memory (FA)	False alarms (%)	0.049
TMS	10	TMS (1)	MEP amplitude mean (0–90 min) v baseline	0.188
	11	TMS (2)	MEP amplitude mean (0–30 min) v baseline	0.105
	12	TMS (3)	MEP amplitude mean (30–90 min) v baseline	0.263
cognition (CANTAB)	13	CANTAB: VRM (1)	Verbal recognition memory, free recall total correct	0.011
	14	CANTAB: VRM (2)	VRM recognition (immediate)	0.014
	15	CANTAB: VRM (3)	VRM recognition (delayed)	0.155
	16	CANTAB: PAL (1)	Paired associates learning, total errors (6 shapes)	0.076
	17	CANTAB: PAL (2)	PAL total errors (8 shapes)	0.186
	18	CANTAB: PAL (3)	PAL total errors (adjusted)	0.101
	19	CANTAB: CRT	Choice reaction time, mean latency	0.211
	20	CANTAB: IED (1)	Intra/extra dimensional set shifting, EDS errors	0.063
	21	CANTAB: IED (2)	IED pre-ED errors	0.127
	22	CANTAB: IED (3)	IED stages completed	0.140
	23	CANTAB: RVP	Raid visual information processing, A-prime	0.079
	24	CANTAB: SRT	Simple reaction time, mean latency	0.071
	25	CANTAB: SWM (1)	Spatial working memory, between errors (6 boxes)	0.018
	26	CANTAB: SWM (2)	SWM between errors (8 boxes)	0.101
	27	CANTAB: SWM (3)	SWM within errors (6 boxes)	0.032
	28	CANTAB: SWM (4)	SWM within errors (8 boxes)	0.032
VMA	29	VMA (1)	adapt mean angular error (blocks 2–10)	0.042
	30	VMA (2)	adapt mean ang error (blocks 11–19)	0.035
	31	VMA (3)	de-adapt mean ang error (blocks 2–10)	0.071
	32	VMA (4)	de-adapt mean ang error (blocks 11–19)	0.084
FLE	33	FLE (1)	conditioning SCR to NS	0.226
	34	FLE (2)	conditioning SCR to CS	0.276
	35	FLE (3)	extinction SCR to CS	0.090
	36	FLE (4)	extinct-retention SCR to CS	0.076
EEG	37	EEG: MMN amplitude	MMN difference wave amplitude	0.075
	38	EEG: MMN latency	MMN difference wave latency	0.080
	39	EEG: Resting (1)	Abs power (cent,alpha)	0.260
	40	EEG: Resting (2)	Abs power (cent,delta)	0.112
	41	EEG: Resting (3)	Abs power (cent,theta)	0.342
	42	EEG: Resting (4)	Abs power (front,alpha)	0.246
	43	EEG: Resting (5)	Abs power (front,delta)	0.062
	44	EEG: Resting (6)	Abs power (front,theta)	0.342
	45	EEG: Resting (7)	Abs power (par-occ,alpha)	0.169
	46	EEG: Resting (8)	Abs power (par-occ,delta)	0.137
	47	EEG: Resting (9)	Abs power (par-occ,theta)	0.337
	48	EEG: Resting (10)	Abs power (temp,alpha)	0.220
	49	EEG: Resting (11)	Abs power (temp,delta)	0.140
	50	EEG: Resting (12)	Abs power (temp,theta)	0.367
	51	EEG: aLTP (1)	aLTP post-tetanae mean v baseline N1 amplitude	0.003
	52	EEG: aLTP (2)	aLTP post-tetanae mean v baseline N1-P2 amp	0.131
	53	EEG: P3a amplitude	P3a peak amplitude	0.009
	54	EEG: P3a latency	P3a peak latency	0.085
	55	EEG: P3b amplitude	P3b AUC	0.047
	56	EEG: P3b latency	P3b peak latency	0.086
	57	EEG: PE-slowing	ERN: PE-slowing	0.250
	58	EEG: ERN amplitude	ERN: amplitude	0.150
	59	EEG: ERN-error ampitude	ERN: ampitude-error	0.097
	60	EEG: ERN latency	ERN: latency	0.070
	61	EEG: ERN ERSP	ERN: Event-related spectral perturbation	0.290
	62	EEG: ERN-error ERSP	ERN: ERSP-error	0.290
	63	EEG: ERN PLF	ERN: Phase-locking factor	0.260
	64	EEG: ERN PLF-error	ERN: PLF-error	0.400
VBM	65	VBM: LT ACC	Grey matter volume (LT Anterior cingulate)	0.263
	66	VBM: LB ACC	Grey matter volume (LB Anterior cingulate)	0.158
	67	VBM: RT ACC	Grey matter volume (RT Anterior cingulate)	0.206
	68	VBM: RB ACC	Grey matter volume (RB Anterior cingulate)	0.183
	69	VBM: Anterior cingulate	DTI: Anterior cingulate grey matter volume	0.310
	70	VBM: Prefrontal cortex	DTI: Prefrontal cortex grey matter volume	0.220
DTI	71	DTI: L Arcuate	Median FA (L Arcuate)	0.198
	72	DTI: R Arcuate	Median FA (R Arcuate)	0.096
	73	DTI: L Cingulum	Median FA (L Cingulum)	0.170
	74	DTI: R Cingulum	Median FA (R Cingulum)	0.192
	75	DTI: Fornix	Median FA (Fornix)	0.096
	76	DTI: L ILF	Median FA (L ILF)	0.208
	77	DTI: R ILF	Median FA (R ILF)	0.249
	78	DTI: Splenium	Median FA (Splenium)	0.199
	79	DTI: L Uncinate	Median FA (L Uncinate)	0.099
	80	DTI: R Uncinate	Median FA (R Uncinate)	0.008

Further details and p-values are reported elsewhere, see main text. Unresolved acronyms: TMS: Trans-cranial magnetic stimulation; MEP: Motor evoked potentials; EDS:Extra-dimensional stage; pre-ED: Prior to the extra-dimesional shift; VMA: Visuomotor Association; FLE: Fear Learning and Extinction; SCR: Skin conductance response; NS: Non-conditioned stimuli; CS: Conditioned Stimuli; MMN: Mismatch negativity; aLTP: auditory long-term potentiation; PE-slowing: Post-error slowing; ERN: Error-related negativity; VBM: Voxel-based morphometry; LT: Left-top (superior); RB: Right-bottom (inferior); FA: Fractional anisotropy; ILF: Inferior longitudinal Fasciculus.

## Discussion

In this study we aimed to develop and validate markers of synaptic activity that are sensitive to BDNF-related synaptic plasticity and could be utilised in clinical trials to test the efficacy of “synaptogenic” therapies in development for neurodegenerative disorders. Taking advantage of the *BDNF* Val66Met polymorphism in humans, we examined the effects of the polymorphism on brain activity and behaviour in healthy subjects using a variety of methods including fMRI, MRI, tDCS, EEG/ERP and behavioral testing. The objective of the study was twofold. First, we tested potential biomarkers of synaptic functioning which have previously been associated with the *BDNF* Val66Met polymorphism. Replication of significant markers will serve us in establishing the most sensitive and reliable tools for future research into ‘synapse repair’ strategies in the prevention and treatment of neurodegenerative and psychiatric disorders utilizing BDNF. Second, we systematically tested the effect of ’met allele load’ across all the significant endpoints in order to reveal whether the relatively rare met homozygotism (<5%) was responsible for the great variability in findings in the literature. In this report we primarily focus on the electrophysiological markers of synaptic activity as some of these markers have been found to be the most sensitive to group differences associated with the genetic polymorphism. However, comparisons were also made with markers in terms of effect size to highlight the greater sensitivity of some of the electrophysiological markers. The detail of the findings on the other markers will be reported and published elsewhere [69], [87].

Across the different platforms and various endpoints, some of the electrophysiological measurements proved to be the most sensitive markers of synaptic functioning affected by the *BDNF* Val66Met polymorphism (as illustrated in **figure 7**). Met carriers showed decreased neural activity and neural synchrony in response to errors (delta band power and PLF), showed decreased post-error slowing, showed increased low frequency activity during resting compared to Val homozygotes. Although similar corrections are not commonly done in neuroscience research, it is interesting to note that the ERN phase locking factor, the resting state EEG theta power measures, and the anterior cingulate grey matter volume remain significant after correction of multiplicity; although, some of the endpoints (behavioural post-error slowing (i.e. number 57 in **Figure 7**) and ERN delta power (number 61 and 62)) do not remain significant (see **Figure 7**). The ERN findings replicate a previous study by Beste et al. [49]. Specifically, we have replicated that Met carriers showed reduced or absent post-error slowing in a flanker-task. The reduced or lacking post-error slowing is suggestive of deficiencies in behavioural adaptation following the commission of errors in Met carriers. We have also replicated that the power and phase-locking in the delta frequency band following errors was reduced in Met carriers, compared to Val/Val subjects. These results signify the altered functioning of the neuronal network subserving error monitoring and behavioural adaptation in Met carriers [49]. Furthermore, in the latter study, phase-locking in the delta band was shown to predict post-error slowing and the authors concluded that stronger neural activity and synchronization mechanisms are associated with elevated behavioural adaptation [49], [59]. In our data, neural dynamics in the delta frequency band also predicted behavioural post-error slowing. However, in contrast to the previous findings [49], we observed that delta power, and not phase-locking, predicted post-error slowing. The reason for this discrepancy is unclear, but may be due to the unavoidable variability and noise inherent to different neuroscience laboratories and to datasets drawn from different groups. But importantly, reduced post-error slowing, reduced delta band power, and reduced delta band phase-locking in Met carriers has now been consistently observed suggesting that the *BDNF* Val66Met polymorphism modulates the functioning of the neural network supporting error processing and behavioural adaptation. Interestingly, abnormalities in ERN have also been reported in neurodegenerative disorders including basal ganglia disorders (Parkinson's and Huntington's disease; [88], [89], [90], [91], [9]. Hence ERN may represent both a BDNF and disease relevant marker that could be used to monitor changes in synaptic activity in clinical trials.

In addition, accompanying the alterations in neuronal functioning, significant differences in the structure within this network have also been shown. Reduced grey matter volume in the anterior cingulate has been previously reported in Met carriers [31], [92]. As can be seen in **Figure 7**, ACC grey matter volume was among the strongest markers sensitive to the *BDNF* polymorphism. Furthermore, and more interestingly, ACC grey matter volume significantly predicted post-error slowing in the flanker task in Met/Met subjects (and in the Met carriers combined), but not in the Val/Val participants. The greater the grey matter loss, the less the post-error slowing, hence less the behavioural adaptation following errors. This finding provides a clear link between structure and function involved in error processing and monitoring behaviour. However, although both a structural measure (ACC GM volume) and a functional measure (delta band power) predict behavioural adaptation following errors, ACC GM volume and delta band power were not associated with each other. The lack of association could be explained as follows. Among the several structural components involved in the error processing network, ACC is only one, and delta band EEG activity is a measure of the summation of the activity of more than one structure in the brain. Hence, it is possible that ACC GM volume in itself is not sufficient to explain delta power changes in the flanker task.

Resting state EEG oscillations also emerged as markers that are sensitive to the *BDNF* Val66Met polymorphism. An earlier study [47] demonstrated increased slow-wave activity (theta, delta frequency bands; 1.5–3.5 Hz and 4–7.5 Hz) and decreased fast-wave activity (alpha frequency band; 8–13 Hz) in Met/Met homozygotes over the whole scalp. We have also found increased slow-wave activity (theta frequency band) in Met carriers when compared to Val/Val homozygotes, over the whole head. Increases in slow frequency oscillatory power has been though to reflect inhibitory synaptic transmission within the cortico-thalamical circuits [68]. Imbalances of the cortical excitatory and inhibitory mechanisms, reflected by altered EEG oscillations, have been observed in neurodegenerative and psychiatric disorders. For example, a shift in power from higher frequencies towards lower frequencies has been found in Alzheimer's disease [93] and in depression [94]. Since BDNF has been shown to regulate excitatory and inhibitory activity [5], [48], the met allele, which has been showed to be associated with suboptimal activity-dependent BDNF secretion in the brain [27], [28], could lead to an imbalance in excitatory and inhibitory neural activity.

In comparison to ERN and resting EEG, we found no effect of the *BDNF* polymorphism on other electrophysiological markers including MMN and P300. Although MMN is a commonly used electrophysiological marker in studies investigating various psychiatric and neurodegenerative disorders, such as schizophrenia, Alzheimer's and Parkinson's disease (for reviews see [95], [96]), to our best knowledge MMN has not yet been investigated as a function of allelic variation in *BDNF* gene polymorphisms. The P300 ERP component was found to be sensitive to the polymorphism in a previous study [29], but it could not be replicated here. Both the MMN and P300 tap into cognitive processes which are potentially affected by the *BDNF* polymorphism and which certainly are compromised in neurodegenerative disorders. However, as noted earlier, findings regarding attention, memory and executive functioning have been inconclusive [97]. It is possible that the variability of these measures is more similar in healthy met carriers and Val/Val subjects; hence these measures are not consistent across different experiments and populations and are not reliable markers of the polymorphism in healthy participants.

The electrophysiological markers ERN and resting EEG, accompanied with the grey matter volume of the anterior cingulate, were found to be the most sensitive to the *BDNF* polymorphism compared to other markers examined (see **Figure 7** and **Table 2**). Effects sizes ranged from around 0.33 to 0.4 for ERN/EEG, while other endpoints scarcely exceeded an effect size of 0.2. For example, right hippocampal activity measured during a memory task with fMRI is around 0.28; the strongest measures of cortical excitability measured by tDCS or by various behavioural measures of cognition (i.e. CANTAB tasks of attention, working memory, learning, reaction time, and fear learning) are all below 0.28. These findings are reported separately and published elsewhere [69], [87].

In this study we also examined for the first time the effect of ‘met allele load’ in a study that was prospectively designed with approximately equal number of subjects in each genotype group. Previous in vitro and in vivo animal studies have shown met allele dependent effects where two met allele carriers were different from those carrying one met allele in activity dependent BDNF release and synaptogenesis [45], [98], [99], but the functional effects of met allele load has not been systematically tested in humans. In this study we observed in most cases no evidence for a met load effect with the findings best explained by the met-dominant model; the exceptions from this model were the behavioural results (post-error slowing) and grey matter volume reductions in the ACC and PFC, where a significant linear trend, i.e. significant stepwise effect of ‘met-load’, emerged. It is possible that impact of the met allele on brain structure and/or function may be dependent on the level of network complexity with greater effects on more explicit measures (i.e. involving multiple brain areas to perform a behavioural process), compared to implicit process (i.e. ERN and resting EEG). This may explain the met allele load effect observed on ERN related behavioural adaption and not the error related brain activity. However findings from this study suggest that impairments in synaptic activity could be measured in Met carriers and having one or two copies of the allele does not impact on the functional impairments.

There are some methodological factors that warrant discussion. First we observed no significant effect of the polymorphism on ERN amplitude (although a slight difference is visible in **Figure 2**), while the effect was significant in the trial-by-trial power and phase-locking data. A possible explanation for this discrepancy is that ERP waveforms are the summation of signals across several trials, hence peaks which vary in their timing from trial to trial are prone to cancellation by the averaging procedure. Meanwhile, the trial-by-trial data can be more sensitive since there is no information loss due to averaging across trials. Second, we did not measure a number of other ERPs relevant to neurodegenerative disorders (sensory evoked components, for example) and hence it is possible that there are other EEG markers that may be sensitive to the *BDNF* polymorphism. Finally we examined the impact of the polymorphism at a single time point and it is possible that the polymorphism may have greater effects on various markers when examined longitudinally. Indeed, we have shown larger effects of the *BDNF* polymorphism on declarative memory and hippocampal volume loss when decline is examined over time (i.e. 36 months) than at a single time point as reported in all previous studies [34].

In summary, using the *BDNF* val66met polymorphism, we have identified several electrophysiological markers that could sensitively and reliably measure synaptic changes in the human brain. The electrophysiological markers including ERN and resting EEG theta power could be used as BDNF sensitive functional markers in early clinical development to examine target engagement or drug related efficacy of synaptic repair therapies. These findings also provide some evidence for the successful use of a common genetic polymorphisms in biomarker development.

## Supporting Information

Figure S1 **Schematic representation of the Flanker task and stimuli.**
(TIF)

File S1 **Supporting Tables. Table S1. Educational status of subjects. Table S2. Absolute EEG power values. Table S3. Mean (and standard error) values and statistics for the non-significant EEG endpoints.**
(DOCX)
