# Supplementary figures and images for: Identification of BDNF Sensitive Electrophysiological Markers of Synaptic Activity and Their Structural Correlates in Healthy Subjects Using a Genetic Approach Utilizing the Functional BDNF Val66Met Polymorphism
Source: PLoS One. 2014 Apr 23;9(4):e95558. doi: 10.1371/journal.pone.0095558 (PMC3997566; doi:10.1371/journal.pone.0095558)

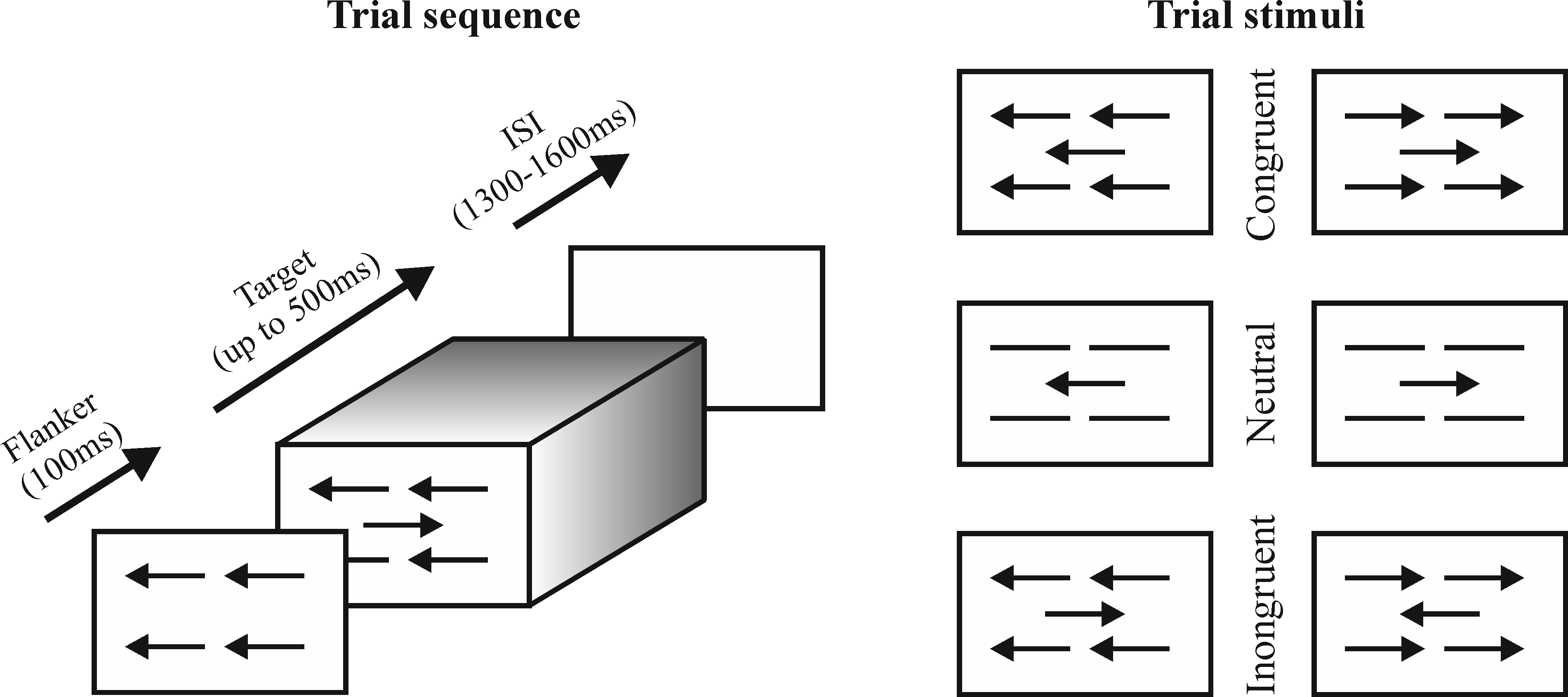

Supplement: Figure S1 — Schematic representation of the Flanker task and stimuli. (TIF) [file pone.0095558.s001.tif]
