# Supplementary material for: Identification of BDNF Sensitive Electrophysiological Markers of Synaptic Activity and Their Structural Correlates in Healthy Subjects Using a Genetic Approach Utilizing the Functional BDNF Val66Met Polymorphism
Source: PLoS One. 2014 Apr 23;9(4):e95558. doi: 10.1371/journal.pone.0095558 (PMC3997566; doi:10.1371/journal.pone.0095558)
Supplement: File S1 — Supporting Tables. Table S1. Educational status of subjects. Table S2. Absolute EEG power values. Table S3. Mean (and standard error) values and statistics for the non-significant EEG endpoints. (DOCX) [file pone.0095558.s002.docx]

**Supporting Information**

Table S1: Educational status of subjects.

| Group | No qualification | CSE/GCE/GCSE | A level | Vocational | Degree |
| --- | --- | --- | --- | --- | --- |
| Met/Met | 1 | 3 | 4 | 5 | 7 |
| Val/Met | 2 | 5 | 2 | 4 | 7 |
| Val/Val | 1 | 5 | 2 | 5 | 7 |

Table S2: Absolute EEG power values. EEG power and corresponding statistics (met-dominant model) for topographic regions and frequency bands. Significant Region/ frequency bands are marked with bold italic typesetting.

| Region | Frequency band | Mean (SE) | |  | |  |  | | Statistics | |  |
| --- | --- | --- | --- | --- | --- | --- | --- | --- | --- | --- | --- |
|  |  | Met/Met | Val/Met | | Met-carriers | | | Val/Val | F value | p value | |
| Frontal | Delta | 0.49 (.03) | 0.5 (.018) | | 0.49 (.025) | | | 0.47 (.026) | 0.63 | 0.43 | |
|  | ***Theta**** | 0.33 (.028) | 0.41 (.029) | | 0.37 (.029) | | | 0.31 (.029) | 4.92 | ***0.03*** | |
|  | Alpha | 0.27 (.018) | 0.31 (.027) | | 0.29 (.023) | | | 0.25 (.016) | 2.22 | 0.14 | |
|  | Beta | 0.14 (.091) | 0.14 (.012) | | 0.14 (.01) | | | 0.12 (.006) | 1.74 | 0.19 | |
|  | Gamma | 0.05 (.008) | 0.04 (.005) | | 0.05 (.007) | | | 0.05 (.006) | 0.00 | 1.00 | |
| Central | Delta | 0.31 (.024) | 0.31 (.012) | | 0.31 (.018) | | | 0.28 (.015) | 2.25 | 0.14 | |
|  | ***Theta**** | 0.26 (.023) | 0.3 (.024) | | 0.28 (.024) | | | 0.22 (.021) | 6.03 | ***0.02*** | |
|  | Alpha | 0.24 (.019) | 0.26 (.022) | | 0.25 (.02) | | | 0.21 (.015) | 2.85 | 0.10 | |
|  | Beta | 0.11 (.008) | 0.11 (.008) | | 0.11 (.008) | | | 0.1 (.005) | 2.11 | 0.15 | |
|  | Gamma | 0.03 (.002) | 0.03 (.003) | | 0.03 (.002) | | | 0.03 (.002) | 0.30 | 0.58 | |
| Temporal | Delta | 0.69 (.047) | 0.73 (.034) | | 0.71 (.04) | | | 0.66 (.038) | 2.58 | 0.11 | |
|  | ***Theta**** | 0.67 (.072) | 0.81 (.069) | | 0.74 (.07) | | | 0.57 (.046) | 7.83 | ***0.01*** | |
|  | Alpha | 0.56 (.048) | 0.61 (.053) | | 0.58 (.05) | | | 0.51 (.04) | 1.78 | 0.19 | |
|  | Beta | 0.25 (.019) | 0.24 (.018) | | 0.24 (.018) | | | 0.21 (.01) | 3.29 | 0.08 | |
|  | Gamma | 0.08 (.01) | 0.07 (.005) | | 0.08 (.008) | | | 0.07 (.007) | 0.41 | 0.52 | |
| Parieto-occ | Delta | 0.49 (.034) | 0.54 (.023) | | 0.51 (.029) | | | 0.48 (.028) | 2.96 | 0.09 | |
|  | ***Theta**** | 0.52 (.058) | 0.65 (.055) | | 0.59 (.057) | | | 0.47 (.045) | 6.10 | ***0.02*** | |
|  | Alpha | 0.49 (.049) | 0.57 (.052) | | 0.53 (.05) | | | 0.49 (.048) | 0.52 | 0.47 | |
|  | Beta | 0.19 (0.15) | 0.2 (.015) | | 0.19 (.015) | | | 0.17 (.008) | 3.12 | 0.08 | |
|  | Gamma | 0.05 (.004) | 0.05 (.003) | | 0.05 (.004) | | | 0.04 (.004) | 0.66 | 0.42 | |

Table S3: Mean (and standard error) values and statistics for the non-significant EEG endpoints.

| Component | Measure | Mean (SE) |  | |  | |  | | Statistics | |  |
| --- | --- | --- | --- | --- | --- | --- | --- | --- | --- | --- | --- |
|  |  | Met/Met | | Val/Met | | Met-carriers | | Val/Val | F value | p value | |
| P300 (P3b) | amplitude (μV) | 4.58 (.7) | | 4.85 (.63) | | 4.73 (.47) | | 4.1 (.62) | 0.64 | 0.43 | |
|  | latency (ms) | 457.6 (14.7) | | 448 (13.3) | | 452.3 (9.8) | | 442.2 (13) | 0.38 | 0.54 | |
| P3a | amplitude (μV) | 13.8 (1.5) | | 14.2 (1.4) | | 14 (1) | | 14.5 (1.4) | 0.07 | 0.79 | |
|  | latency (ms) | 330.5 (6.8) | | 326.8 (6.1) | | 328.5 (4.5) | | 322.6 (6) | 0.61 | 0.44 | |
| MMN | amplitude (μV) | -1.67 (.18) | | -1.42 **(**.17**)** | | -1.54 **(**.12**)** | | -1.38 **(**.16**)** | 0.62 | 0.44 | |
|  | latency (ms) | 177.5 (5.1) | | 187.9 (4.9) | | 182.9 (3.6) | | 186.1 (4.7) | 0.28 | 0.60 | |
